# The Enzyme and the cDNA Sequence of a Thermolabile and Double-Strand Specific DNase from Northern Shrimps (*Pandalus borealis*)

**DOI:** 10.1371/journal.pone.0010295

**Published:** 2010-04-22

**Authors:** Inge W. Nilsen, Kersti Øverbø, Linda Jensen Havdalen, Morten Elde, Dag Rune Gjellesvik, Olav Lanes

**Affiliations:** 1 Nofima Marin, Tromsø, Norway; 2 Marine Biochemicals, Tromsø, Norway; Institute of Evolutionary Biology (CSIC-UPF), Spain

## Abstract

**Background:**

We have previously isolated a thermolabile nuclease specific for double-stranded DNA from industrial processing water of Northern shrimps (*Pandalus borealis*) and developed an application of the enzyme in removal of contaminating DNA in PCR-related technologies.

**Methodology/Principal Findings:**

A 43 kDa nuclease with a high specific activity of hydrolysing linear as well as circular forms of DNA was purified from hepatopancreas of Northern shrimp (*Pandalus borealis*). The enzyme displayed a substrate preference that was shifted from exclusively double-stranded DNA in the presence of magnesium to also encompass significant activity against single-stranded DNA when calcium was added. No activity against RNA was detected. Although originating from a cold-environment animal, the shrimp DNase has only minor low-temperature activity. Still, the enzyme was irreversibly inactivated by moderate heating with a half-life of 1 min at 65°C. The purified protein was partly sequenced and derived oligonucleotides were used to prime amplification of the encoding cDNA. This cDNA sequence revealed an open reading frame encoding a 404 amino acid protein containing a signal peptide. By sequence similarity the enzyme is predicted to belong to a family of DNA/RNA non-specific nucleases even though this shrimp DNase lacks RNase activity and is highly double-strand specific in some respects. These features are in agreement with those previously established for endonucleases classified as similar to the Kamchatka crab duplex-specific nuclease (Par_DSN). Sequence comparisons and phylogenetic analyses confirmed that the Northern shrimp nuclease resembles the Par_DSN-like nucleases and displays a more distant relationship to the *Serratia* family of nucleases.

**Conclusions/Significance:**

The shrimp nuclease contains enzyme activity that may be controlled by temperature or buffer compositions. The double-stranded DNA specificity, as well as the thermolabile feature, strengthens its potential for *in vitro* applications.

## Introduction

Modern biotechnology tools comprise the use of enzymes originating from various sources. Particularly useful are specialized enzymes from organisms, including marine animals, living under more or less “extreme” conditions. Among the most commonly used are enzymes with unusual heat stability or instability such as the thermophilic Taq polymerase [1] and its equivalents for PCR amplification [2], and the thermolabile shrimp alkaline phosphatase [3], [4] for PCR “clean-up” prior to DNA sequencing [5] or for DNA dephosphorylation in cloning [6].

Enzymes that are capable of cleaving or hydrolysing/degrading nucleic acids are referred to as nucleases. Best known is probably DNase I that is found in a variety of sources. It preferably degrades double-stranded (ds) DNA, but activity against single-stranded (ss) DNA has also been indicated [7]. A nuclease from the *Penaeus japonicus* shrimp was the first nuclease characterized and sequenced from Arthropoda [8] which showed sequence similarity to the *Serratia marcescens*-like unspecific nucleases [9]. No substrate specificity was revealed for the *P. japonicus* nuclease, except for a potential small intrinsic RNase activity. Only a few nucleases with an exclusive dsDNA activity and no sequence-specificity have been reported, and aquatic as well as terrestrial invertebrates have been reported to be sources of such particular dsDNases [10]–[12]. The king crab, *Paralithodes camtschaticus*, harbors a nuclease with exclusive preference for dsDNA [13] or DNA-RNA duplexes and no ssDNase or RNase activity [10]. This crustacean enzyme is by nature tolerant to high temperatures [14] but a relatively thermolabile version of the dsDNase was constructed by random mutagenesis as very recently reported [15]. Although not reporting similar data about enzyme activity as a function of temperature, yet another nuclease displaying exclusive activity against dsDNA and lack of RNase activity was found secreted in females of the mosquito *Culex quiquefasciatus* [12].

We have previously isolated a thermolabile nuclease specific for double-stranded DNA from industrial processing water of Northern shrimps (*Pandalus borealis*) and developed an application of the enzyme in removal of contaminating DNA in PCR-related technologies [11]. Here we report the isolation and characterization of this dsDNase from Northern shrimp hepatopancreas as well as the sequence analysis of the gene and its derived protein sequence.

## Materials and Methods

### Materials

Northern shrimps (*Pandalus borealis*) were frozen immediately after collection by a local fishing vessel. Chromatography columns and FPLC equipment were products of GE Healthcare. Calf thymus DNA (D-1501) and plasmid DNA were purchased from Sigma and GE Healthcare, respectively. Pre-casted Novex NuPAGE gels and the TOPO TA cloning Kit were delivered by Invitrogen. SMART cDNA library construction kit and Marathon cDNA amplification Kit were products of Clontech. General reagents and chemicals were of analytical grade from various suppliers. RNase free bovine pancreatic DNase I was purchased from Sigma, while King crab nuclease (DSN) and recombinant shrimp nuclease were provided from Evrogen (Russia) and Marine Biochemicals (Norway), respectively. Labelled oligonucleotides were purchased from MWG/Eurofins.

### Nuclease activity determination

DNase activities were measured by both the Kunitz assay, and a modified version of Kunitz [16], [17]. The latter, in which end-point readings are performed, includes running the assay at pH 8.0 and stopping the reaction by addition of 0.5 volumes of 12% HClO_4_, removing precipitated material by centrifugation, and reading the absorbance in the supernatant at 260 nm [17]. For both assays, one unit of DNase activity corresponds to an increase in absorbance of 0.001/min. Substrate-dependent activities were detected using an in-house PCR product, supercoiled plasmids (pBR322 or pGEM-3Zf), calf thymus DNA (double-stranded or heat-denatured) or total RNA isolated from HeLa cells.

### Purification of shrimp nuclease

Digestive glands were isolated from shrimps while still frozen and kept on ice. Extraction of the fragile hepatopancreas tissues took place by 18 h of stirring in four volumes of extraction buffer (50 mM sodium acetate, pH 5.5). The crude extract was clarified by centrifugation (13,000 *g*, 20 min) and then dialysed against 5 mM Tris-HCl, pH 7.4. After addition of MgCl_2_ to a concentration of 5 mM, a concentration kept through-out the purification process, the dialysed extract was centrifuged once more, and this final extract was subjected to serial steps of chromatography with adequate dialyses between each step (all steps performed at 12°C).

The extract was applied on Q Sepharose FF pre-equilibrated with buffer A (50 mM Tris-HCl, pH 7.4), and bound materials were eluted by running a NaCl-gradient (0→1 M) in buffer A. Various nuclease activities were detected after separation, and the double-strand (ds) specific activity addressed in this report eluted at 0.4 M NaCl. After adjusting salt concentration to 3 M, the eluate was subjected to Phenyl Sepharose CL-4B chromatography employing a reverse NaCl-gradient (2→0 M) in buffer A, and nuclease activity was detected in the flow-through and washing fractions only. Next, the nuclease-containing material was subjected to size-exclusion chromatography employing Sephacryl S-200 HR and the previously defined buffer A with the addition of 0.15 M NaCl. Nuclease activity was measured in two neighbouring fractions corresponding to molecular weights of 45-50 kDa as judged by the use of a set of protein standards in separate experiments. The two fractions were pooled and applied to Red Sepharose CL-6B. The column was washed with buffer A after which the NaCl concentration was increased in steps up to 1.5 M and the dsDNase was eluted at 0.5 M NaCl. Subsequent freeze-drying and resuspension of the protein was finally followed by desalting on a PD-10 column using a 20 fold dilution of buffer A.

### Shrimp nuclease activity characterizations

For visualisation and *in-gel* discrimination between activity against ss- and dsDNA, the PCR product or calf thymus DNA was boiled for 10 minutes and immediately placed on ice in order to retain a significant portion of the DNA denatured. These PCR product substrates were incubated with 0.2 units nuclease in a common PCR buffer for 5 minutes at room-temperature. At the end of incubation, the reactions were analysed by standard agarose gel electrophoresis. To examine potential RNase activity in the shrimp nuclease preparation, the enzyme was incubated at 37°C for 30 min with 1.2 µg of total RNA from HeLa cells in a buffer containing divalent cations (MgCl_2_ or MgCl_2_ + CaCl_2_).

A cutting-religation experiment was done by partly hydrolysing 4 µg plasmid DNA using 0.01 U purified shrimp dsDNase in 50 µl total volume (20 mM Tris/HCl, pH 8, 5 mM MgCl_2_). The reaction mix was incubated at 37°C for 10 minutes and thereafter extracted once with phenol/chlorophorm and ethanol precipitated. Digested DNA was resuspended in 10 µl TE-buffer and half of the suspension was included in a ligation reaction (in a total volume of 10 µl) containing 2 U T4-DNA ligase and buffer (USB). The ligation mixture was incubated at 16°C overnight before analysis by agarose gel electrophoresis. Any form of DNA or RNA in gels was detected by UV-illumination after staining with ethidium bromide or GelStar ® (Lonza).

DNA substrate selectivity was further investigated employing labelled ss- or ds-oligonucleotides in a fluorescence resonance energy transfer (FRET) assay. As substrate for ssDNA a 15 nt dual labelled oligonucleotide was used (5′-FAM - CGC CAT CGG AGG TTC- BHQ1-3′ ), whereas a dsDNA substrate was prepared by mixing equimolar amounts of two complementary oligonucleotides labelled with FAM at the 5′-end or with a BHQ1-quencher in the 3′-end, respectively (5′-FAM - CGC CAT CGG AGG TTC -3′/5′- GAA CCT CCG ATG GCG BHQ1 -3′ -). The cleavage reactions were carried out in a total volume of 200 µl, containing 0.2 µM substrate DNA in a buffer of 25 mM Tris/HCl, pH 8, 5 mM MgCl_2_ and using 0.025-0.1 U DNase for measuring dsDNA cleavage and 1–100 U enzyme for ssDNA cleavage. Enzyme activities as initial rates of fluorescent intensity increase at 37°C were determined using a Victor^3^ 1420 Multilabel Counter (Perkin Elmer).

Temperature stability was tested by adding 16 U shrimp nuclease to a 20 mM Tris/HCl, pH 8.0, 5 mM MgCl_2_ buffer in final volume of 250 µl. The reaction tube was incubated at 65°C and samples were transferred to ice and remaining activity was measured using the endpoint assay.

### SDS-PAGE and protein sequencing

Purity of dsDNase was investigated by standard SDS polyacryalamide gel electrophoresis and subsequent staining (coomassie and silver) of proteins, exploiting the Novex NuPAGE gel system running a 10% Bis-Tris gel. Samples of the apparently pure protein (lyophilizd or in solution) were sent to a commercial service (Eurosequence; Groningen, The Netherlands) and sequence-analysed following the principles of Edman degradation [18] for sequencing of the N-terminus and of selected internal fragments after tryptic digestion.

### Preparation of cDNA from hepatopancreas

mRNA was isolated from 105 mg shrimp hepatopancreas tissue using the Oligotex Direct mRNA Mini Kit (Qiagen) according to the manufacturer instructions. First strand cDNA synthesis by reverse transcription of isolated polyadenylated RNA and amplification of cDNA was performed using the SMART cDNA library construction kit according to the protocol recommendations. The PCR amplification was initiated by 95°C for 1 minute followed by 18 cycles of 95°C for 15 seconds and 68°C for 6 minutes. Enzymes in the reaction mixture was digested by adding 40 µg proteinase K to 50 µl of the amplified cDNA and incubated at 45°C for 45 minutes followed by inactivation of the proteinase by heating at 90°C for 8 min. The cDNA ends were blunted using 15 U of T4 DNA polymerase (Promega) at 16°C for 30 min followed by inactivation at 72°C for 10 min. Finally the volume was adjusted to 100 µl with H_2_O and the cDNA was extracted once with phenol/chloroform before precipitation of DNA by ethanol and resuspension of cDNA in 5 µl TE-buffer.

### Generating a shrimp nuclease gene fragment

Degenerated primers were designed based on the peptide sequences determined from the isolated protein. These primers were used in many combinations in PCR in order to amplify a fragment of the gene. The PCR primer combination that gave a shrimp nuclease gene fragment using the synthesized cDNA library as template was derived from the N-terminal peptide sequence EDCVWDN (5DNase, 5′- GAR GAY TGY GTN TGG GAY AA -3′) and a tryptic internal peptide sequence INNPHIN (K20-R1, 5′- RTT DAT RTG NGG RTT RTT DAT -3′). Amplification was carried out in a final volume of 50 µl containing 10 mM Tris/HCl pH 9.0 (25°C), 50 mM KCl, 0.1% Triton X-100, 0.2 mM dATP, dCTP, dGTP and dTTP, 2.0 µM forward primer (5DNase) and reverse primer (K20-R1) and 1.25 U Taq-polymerase (Promega). Touchdown PCR was done by 94°C for 2 min, 5 cycles of 94°C for 15 sec, 60 → 55°C for 1 min (with a 1°C decrement each cycle) and 72°C for 1.5 min, and 30 cycles of 94°C for 15 sec, 55°C for 1 min and 72°C for 1.5 min using a Biometra TGradient thermocycler. The resulting 1 kbp fragment was finally cloned into a pCRII-TOPO vector and sequenced using Big Dye Terminator Cycle Sequencing Kit and a 3100 Genetic Analyser (ABI).

### Adapter ligation and rapid amplification of cDNA ends (RACE)

Ligation of RACE-adapters to the synthesized cDNA library was performed as described for the Marathon cDNA Amplification Kit followed by incubating for 5 min at 70°C to inactivate the ligase. Prior to the RACE procedure, adapter-ligated cDNA was diluted fifty fold in TE-buffer and denatured by heating at 100°C for 2 min and thereafter placed directly on ice. The sequence analysis of the Northern shrimp nuclease 1 kbp gene fragment was used to design primers for both 3′- and 5′- RACE, creating a small sequence overlap in the two fragments to be generated. Both 3′- and 5′-RACE reactions were run on diluted adapter-ligated cDNA template employing 0.2 µM of gene specific primers DN1F (5′-ATA CGA CAC CCG TGA CCT GGC TGA A-3′) or DN4R (5′-TGT CCT GGG CGA GGC CAA GAT AGA T-3′) in combination with 0.2 µM of the AP1-primer and using the Advantage 2 polymerase Mix and buffer provided in the Marathon kit. RACE reactions were carried out by initial heating at 94°C for 1 min, followed by 5 cycles of 94°C for 15 sec, 66 → 62°C for 30 sec and 72°C for 2 min, and 30 cycles of 94°C for 15 sec, 62°C for 30 sec and 72°C for 2 min. The resulting 1000 and 1100 bp fragments were purified from gel and used as a template in two separate nested PCR using DN2F (5′- CAT GGC ACT GAC CTG ACC GTC TAC AGT - 3′) or DN3R (-5′ GGG GTT TCC GTT GAT GTC TTC AAG CT - 3′) as respective gene specific primers in combination with the AP1 primer as above. These nested amplifications were carried out employing an initial heating at 94°C for 1 min, followed by 25 cycles at 94°C for 15 sec, 68°C for 30 sec and 72°C for 2 min. The PCR products were purified using ExoSAP-IT (USB) and sequenced using Big Dye Terminator Cycle Sequencing Kit and a 3100 Genetic Analyser (ABI). The assembled gene sequence was deposited in GenBank and received the accession number FN554584.

### Results and Discussion

**Figure 1:**
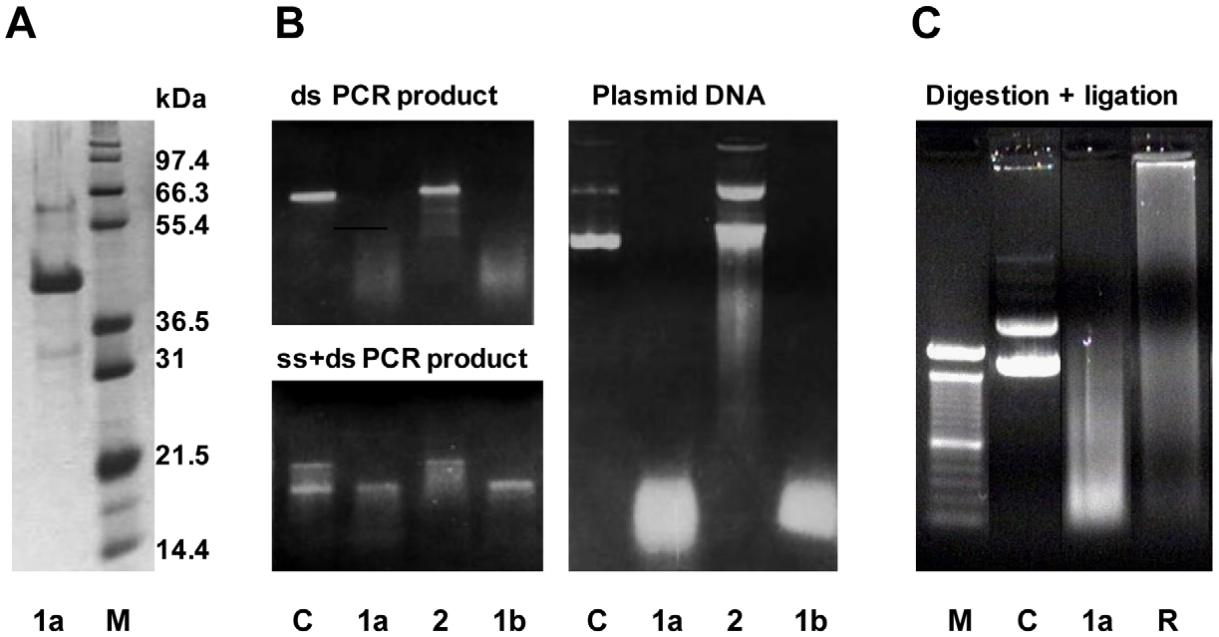
Purified Northern shrimp nuclease and its DNA substrates. The enzyme after SDS-PAGE and silver-staining (**A**), its hydrolyzing activity on different DNA substrates (**B**), and ligation of its digestion products (**C**) as analyzed by agarose gel electrophoresis. Activities of two preparations of the shrimp dsDNase (1a and 1b) and of a shrimp ss nuclease (2) on a dsDNA PCR product, a heat denatured PCR product (ds+ssDNA) and on supercoiled plasmid DNA are shown. *M* defines the lane of protein MW standards and of a 100 bp DNA ladder in the relevant gel types, and lanes *C* represent the respective untreated DNA substrates used. Lane *R* shows the ligated reaction products from dsDNase digested plasmid DNA.

From earlier work we knew that industrial processing water used for thawing of *Pandalus borealis* shrimps contained several nucleases including a thermolabile DNase with a specific activity against double-stranded DNA [11]. In the present study we aimed to characterize functional and molecular aspects of this dsDNase. An extended scheme of purification was followed as described in the methodsection to purify the nuclease from hepatopancreas, a digestive gland of shrimps. The result in Figure 1A shows one major protein after SDS-PAGE and silver staining, from which the molecular weight was determined to 43 kDa. The substrate specificity of the nuclease is demonstrated in Figure 1B since only the double stranded form of a PCR product was degraded, indicating that the nuclease has specificity to dsDNA. Furthermore, the enzyme acts as an endonuclease as judged from its degradation of a covalently closed circular plasmid. It should be noted that other nuclease activities were excluded during purification of the dsDNase, among them a nuclease with specificity against ssDNA. As a control for substrate-specificity, the demonstration of this ss nuclease activity was included in Figure 1B. Partially digestion of plasmid DNA using the shrimp dsDNase produced fragments that were re-ligated by T4 DNA-ligase (Figure 1C), demonstrating that the nucleolytic product of the shrimp nuclease contains free 5′-phosphomonoester and 3′-OH end groups.

**Figure 2:**
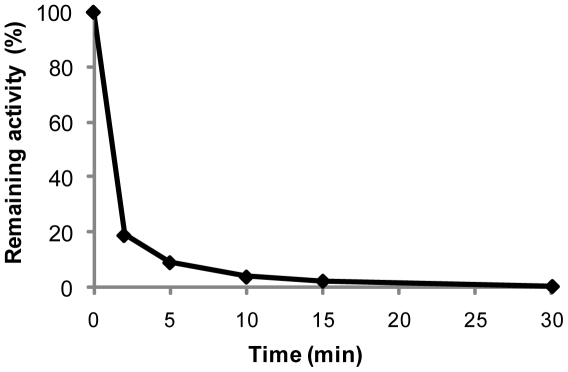
Thermal inactivation. Residual activity of purified Northern shrimp nuclease using the modified Kunitz assay after heating at 65°C for indicated periods of time.

Optimum temperature for this shrimp enzyme was measured to 35–40°C, with a 75% reduction in activity at 25°C and only minor activity at 12°C (results not shown). Thermal stability was determined after incubating the nuclease at various temperatures. Preheating the enzyme above 50°C resulted in a rapid initial inactivation the first 2 minutes, and after 10 min at 65°C more than 95% of the nuclease was inactivated as shown in Figure 2. A similar temperature-sensitivity as seen with shrimp dsDNase was previously described for a DNase I-like nuclease purified from Atlantic cod [19]. This marine vertebrate endonuclease showed activity against native and denatured genomic DNA, and a half-life of 10 min at 50°C with total loss of activity after 10 min at 60°C [20].

**Figure 3:**
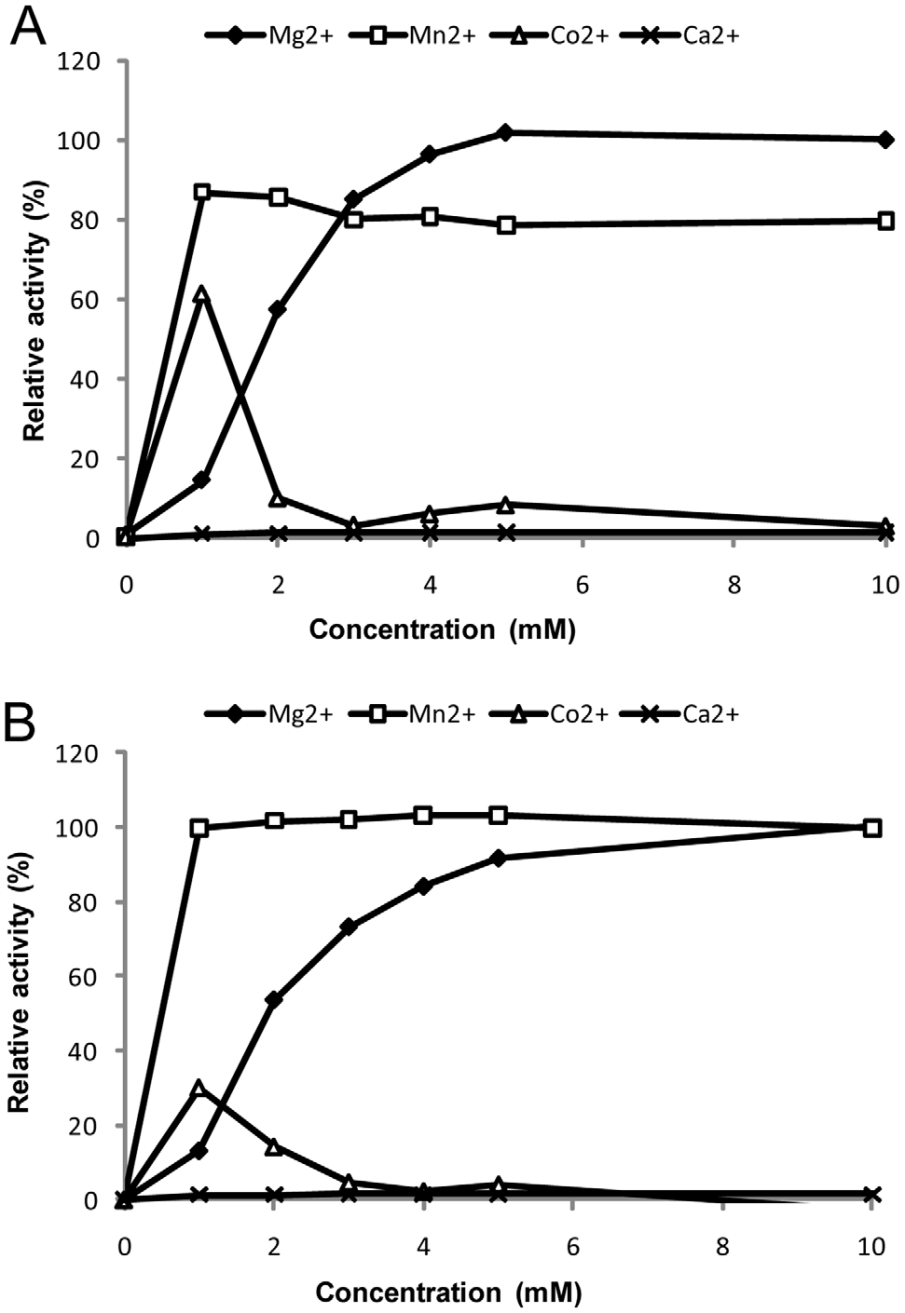
Influence of divalent cations on enzyme activity. Relative activity of native (A) and recombinant (B) shrimp nuclease in presence of various divalent cations. Enzyme activity was measured using the modified Kunitz assay, and one hundred percent activity was set at 10 mM MgCl_2_.

**Figure 4:**
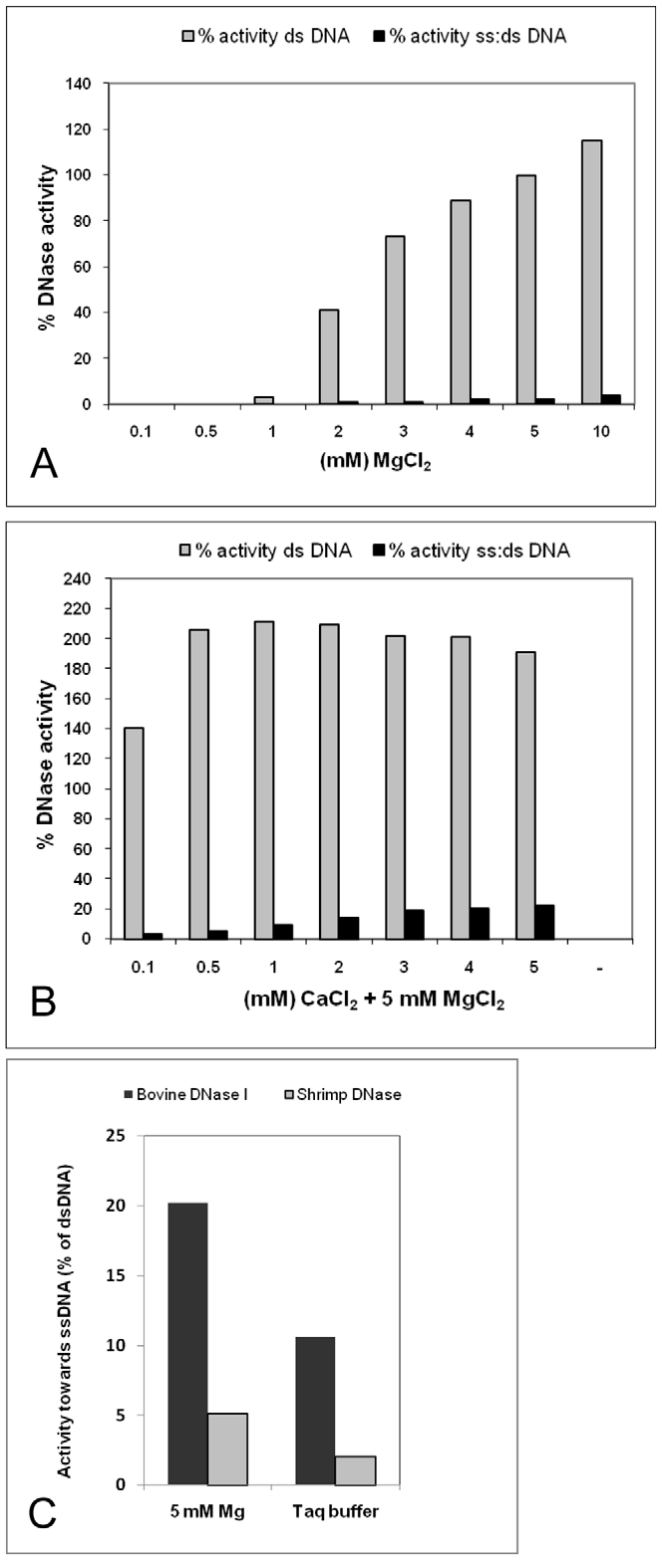
Nuclease activity and specificity against ss- and dsDNA. Relative activity of Northern shrimp nuclease on double stranded (ds) and heat denatured (ss) DNA as a function of divalent cations (**A**: magnesium alone, **B**: magnesium plus calsium) using the modified Kunitz assay. One hundred percent activity was set using dsDNA substrate at 5 mM MgCl_2_. The ss nuclease-activity represents the relative activity on heat-denatured when compared to native ds calf thymus DNA. **C** displays relative activities of bovine pancreatic DNase I and *P. borealis* dsDNase on ssDNA in the described assay buffer or in a conventional Taq-buffer.

The shrimp DNase specific activity on native calf thymus DNA was measured to 1×10^7^ Units/mg at 37°C in the presence of 5 mM MgCl_2_ at pH 8.0 using the endpoint assay [17], and 1.4×10^5^ Units/mg when following the original Kunitz assay method performed at pH 5 [16]. Using the endpoint assay, we found that the enzyme activity at pH 8 was 10 fold higher than at pH 5 and approximately 5 fold higher than at pH 9 (results not shown). The enzyme activity relied on the presence of divalent cations and optimum activity was found using 5–10 mM MgCl_2_ (Fig. 3A). The activity was also stimulated by MnCl_2_ and CoCl_2_, but not by CaCl_2_ alone. Comparison of the activity profile of the native enzyme to the commercially available recombinant version of the enzyme (Figure 3B), revealed similar activity profiles for the divalent cations tested. A decrease in enzyme activity was observed upon addition of more than 10 mM of divalent cations (results not shown). In a separate experiment the magnesium-dependent nuclease activity was tested on genomic DNA with and without prior heat denaturation of the substrate, and the relative activities on these substrates demonstrated that the shrimp DNase has high preference for dsDNA (Figure 4A). Although calcium alone had no positive effect on the nuclease activity, a two-fold increase in dsDNA activity was seen when calcium was added in combination magnesium (Figure 4B). However, this also resulted in a significant change in substrate specificity as introduction of calcium produced an increase in the DNase activity on heat-denatured DNA (Figure 4B). It is expected that heat denaturation of DNA and subsequent rapid cooling will cause some of the DNA to renature into double strand conformation. We thus believe that the very low relative activity on single-stranded DNA (1–5%) of the shrimp dsDNase in the presence of magnesium and absence of calcium actually is a measure for activity on renatured (double stranded) DNA. Surprisingly, many literature sources claim the bovine pancreatic DNase I to be *specific* towards dsDNA instead of having a relative *preference* to dsDNA. To demonstrate this difference, we have included a comparison of the activities from bovine DNase I and our shrimp dsDNase in Figure 4C. Bovine DNase I shows 5 fold higher relative activity to denatured DNA than the shrimp nuclease in the two buffer systems that were tested. In a further examination of nuclease dsDNA substrate selectivity we used synthetic 15 nt labelled oligonucleotides in a FRET assay similar as used for characterizing the crab nuclease [10]. To increase the dynamic range of the dsDNA assay we placed the fluorophore on the 5′-end of the first oligonucleotide strand and the quencher on the 3′-end of the complementary strand, respectively. The substrate specificities of the shrimp nuclease, the crab nuclease [14] and DNase I was compared using this substrate, either as ssDNA (first strand only) or as dsDNA (first and complementary strand). The results from FRET-assays show a relative cleavage ratio of 0.4% on ssDNA compared to dsDNA for the native shrimp dsDNase (Table 1). This single-strand activity was much higher than for the recombinant dsDNase (relative cleavage ratio 0.001) and is an anticipated result of a potential contaminant activity in the native dsDNase preparation. Due to limited available amounts of crab nuclease, we were only able to demonstrate that its relative activity on ssDNA was less than 0.1% of dsDNA as 10 Units Crab nuclease showed no significant ssDNase activity above background. Addition of calcium gave no significant change in the shrimp dsDNase substrate selectivity using this assay (result not shown). The FRET assays demonstrated that DNase I has a much higher ss:ds DNA activity ratio (5%) compared to the shrimp- and crab nucleases, in agreement with the result using denatured genomic DNA.

**Table 1:** Relative activity of various DNases on single- or double-stranded DNA oligonucleotides.

Enzyme	ssDNA vs. dsDNA activity (%)
Native shrimp dsDNase	0.4
Recombinant shrimp dsDNase	0.001
King crab nuclease (DSN)	<0.1
Bovine DNase I	5

**Figure 5:**
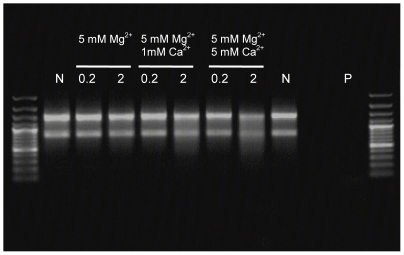
Examination of RNase activity in the shrimp nuclease preparation. Total RNA was incubated with 0.2 or 2 units of enzyme in the presence of only MgCl_2_ or of MgCl_2_ + CaCl_2_. Negative control (N: no enzyme), positive control (P: commercial RNase A).

The calcium-induced shrimp nuclease activity on denatured calf thymus DNA motivated an examination of putative intrinsic RNase activity. The enzyme was incubated with total RNA for an extended period of 30 min after which gel analyses showed no visible RNA degradation in the presence of 5 mM MgCl_2_, but some degradation of the total RNA upon addition of 5 mM MgCl_2_ and CaCl_2_ (Figure 5). Two concentrations of the shrimp DNase was used, of which for comparison the lowest concentration of 0.2 units totally degraded similar amounts of DNA within few minutes as seen in Fig. 1B. A commercial RNase A used as control degraded all the RNA present under the same conditions. We conclude that the dsDNase possesses no (or very low) RNase activity in the absence of Ca^2+^, similar to previous findings in the *P. japonicus* nuclease [8]. The EC classification of the shrimp nuclease thus remains ambiguous, since the data points to a deoxyribose-specific endonuclease with 3′-OH cleavage product with preference for dsDNA (EC 3.1.21.1). On the other hand, if the trace activity on RNA in the presence calcium is an intrinsic property of the enzyme, it could belong in the EC 3.1.30.2 group, although the dsDNA specificity might exclude this classification.

**Figure 6:**
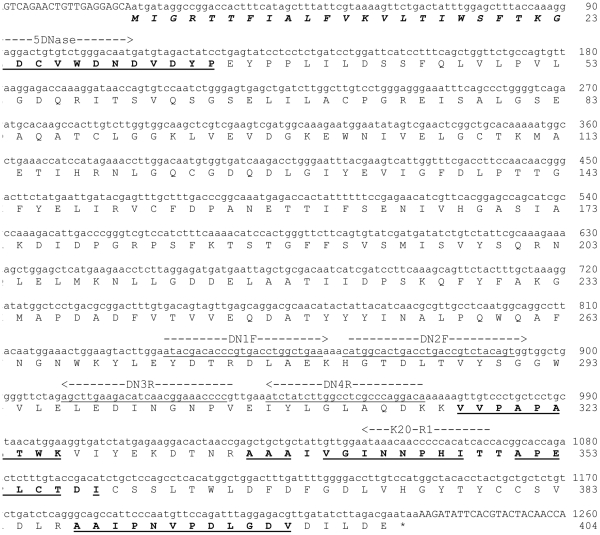
cDNA and amino acid sequence of Northern shrimp dsDNase. Coding sequence is shown in lower letter cases with the derived protein sequence below. A predicted protein signal sequence is highlighted in italics. Location of native protein fragments sequences and their sequence identities compared to cDNA-derived amino acid residues are highlighted and underlined, respectively. Primers used for amplification reactions are indicated above their corresponding cDNA sequence. (GenBank accession no. FN554584).

The purified protein displaying specific and thermolabile dsDNase activity was subjected to sequence analysis. The N-terminal amino acid sequence and partial sequences of three tryptic fragments were identified. Numerous sequence-degenerated primers were used in attempts to amplify the nuclease gene in a cDNA library from the Northern shrimp. A specific PCR product was obtained using a primer combination (5DNase + K20-R1) derived from the N-terminal peptide sequence and from the tryptic internal K20 fragment (see Figure 6). The cDNA predictably encodes a polypeptide sequence in full accordance with three of the four peptide sequences obtained from the native Northern shrimp dsDNase. The ambiguities in the fourth peptide fragment (K20) could be explained by poor peptide sequencing data if minor and otherwise undetected impurities in the protein sample exist, or that there are isoforms of the shrimp nuclease giving ambiguities in some positions. No other cDNA encoding potential enzyme iso- or allelic forms was identified, however, and most likely the obtained gene sequence encodes the dsDNase enzyme that here is purified shrimp hepatopancreas, and previously, from shrimp processing water [11].

The shrimp cDNA sequence, of which a 500 bp 3′-UTR is omitted in Figure 6, contains an open reading frame of 1215 bp encoding a 404 amino acid polypeptide. The SignalP program [21] predicted that residues 1–23 represent a putative signal peptide of the shrimp nuclease. This prediction was in full accordance with our N-terminal native protein sequence that starts with residue 24 in the cDNA-encoded sequence. Using InterProScan [22], we found that the derived shrimp DNase protein sequence matches a family of DNA/RNA non-specific nucleases (IPR001604: Endonuclease_NS/DNA/RNA non-specific endonuclease family). A family-characteristic NUC-domain (IPR020821: NUC/extracellular endonuclease, subunit A domain) was also identified when analyzing the shrimp nuclease sequence using the SMART software tool [23]. This domain includes a histidine found to be essential for activity in the non-specific endonuclease from *Serratia marcecens* [24]. This family of bacterial and eukaryotic endonucleases (EC 3.1.30) acts on both DNA and RNA, cleaves double-stranded and single-stranded nucleic acids at equal rates and requires a divalent ion such as magnesium for their activity. In contrast, no sequence motifs or patterns indicating a relation of the shrimp nuclease to DNase I was identified by any of the employed bioinformatic tools. This was somewhat surprising since (as already discussed) the mode of action and substrate preferences of the shrimp nuclease is much more similar to DNase I than to the DNA/RNA nonspecific nucleases which it shares primary structure similarities.

When compared to public data bank sequence entries by BLAST search [25] the Northern shrimp dsDNase was found to share significant sequence homology (54–65% identity) to a group of marine crustacean (i.e. from crabs and shrimps) and less significantly to insect nucleases (30% identity), all of similar size to our Northern shrimp protein. The referred class of sequences seems to belong to a divergent branch of the DNA/RNA non-specific nucleases, so far only found in Arthropods [26]. These nucleases have an extended NUC-domain as compared to the *Serratia marcesens*-like nucleases, and the NUC domain is suggested to be important for their dsDNA selectivity [26]. However, no structural data have yet been presented to support this hypothesis, and the underlying selectivity for double stranded DNA remains unknown.

**Figure 7:**
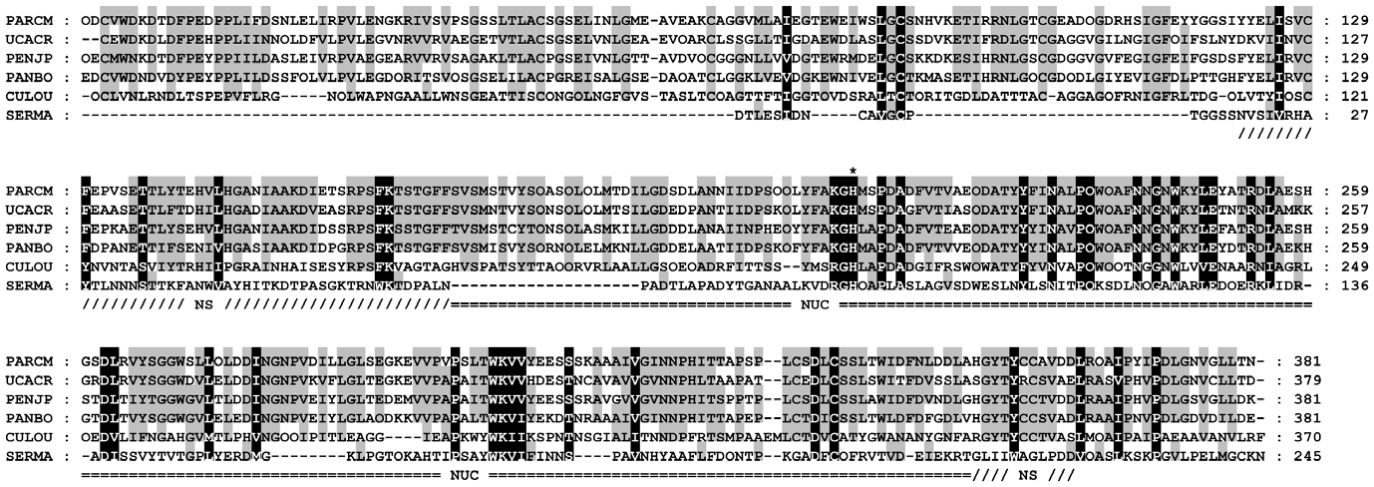
Multisequence alignment of selected NUC endonucleases. Retrived sequences were stripped for signal peptide sequences and aligned using Clustal W2. Amino acids are shaded by degree of conservation (grey for major, black for total). An asterix marks the site of a histidine residue previously reported essential for catalytic activity. Recognised endonuclease NS and NUC domains are indicated according to their occurrence in the *P. borealis* enzyme. The sequences used are from Northern shrimp (PANBO - C9YSL6), Kuruma prawn (PENJP - Q9U5L8), Red king crab (PARCM - B6ZLK3), Fiddler crab (UCACR - Q0GIJ2), Southern house mosquito (CULQU - B0WUW1), and the gram-negative bacterium *Serratia marcescens* (SERMA–P13717).

The alignment (Clustal W2 [27]) to selected sequences of previously studied or predicted NUC endonucleases presented in Figure 7 demonstrates that the Northern shrimp dsDNase carries the larger NUC domain recently defined to be a characteristic of the Par_DSN-like nucleases as opposed to the shorter NUC domain in *Serratia* family of nucleases (SFN) [26]. The most notably feature that separates the crustacean group from other NUC-domain members, is the substitution of the aspartate residue to alanine where the central highly conserved DRGH-motif is changed to AKGH. In Serratia nuclease, the D86 residue has been proposed to participate indirectly in Mg-binding by coordinating N119 [28]. Indeed, the results of Friedhoff et al (1996) [29] show that a D86A mutation in the Serratia nuclease results in an increased requirement for magnesium the nuclease, giving a similar Mg-dependence as shown here for the shrimp DNase.

The shrimp DNase reported here shows a combination of distinct enzymatic properties that in our opinion highlight a potential for use in several *in vitro* applications such as PCR-related techniques and routine work. In particular, it is beneficial that the enzyme activity as well as substrate specificity may be controlled by temperature or buffer compositions (i.e. divalent cations), thus making it convenient for sequential reactions often used in molecular biology.
